# Impact of the Acetaldehyde-Mediated Condensation on the Phenolic Composition and Antioxidant Activity of *Vitis vinifera* L. Cv. Merlot Wine

**DOI:** 10.3390/molecules27092608

**Published:** 2022-04-19

**Authors:** Lingmin Dai, Ke Zhong, Yan Ma, Xiaoqian Cui, Yuhang Sun, Ang Zhang, Guomin Han

**Affiliations:** 1 School of Bioengineering, Qilu University of Technology (Shandong Academy of Sciences), Jinan 250353, China; dailingmin@qlu.edu.cn (L.D.); zk17854117992@163.com (K.Z.); 13287791781@163.com (Y.M.); 17863908085@163.com (X.C.); syhwssy@163.com (Y.S.); 2 Technology Centre of Qinhuangdao Customs, Qinhuangdao 066004, China; zhanganggrape@hotmail.com

**Keywords:** wine, antioxidant, acetaldehyde, aging, monomeric phenolics, polymeric phenolics

## Abstract

Acetaldehyde is a critical reactant on modifying the phenolic profile during red wine aging, suggesting that the acetaldehyde-mediated condensation can be responsible for the variation of antioxidant activity during the aging of this beverage. The present study employs exogenous acetaldehyde at six levels of treatment (7.86 ± 0.10–259.02 ± 4.95 mg/L) before the bottle aging of Merlot wines to encourage phenolic modification. Acetaldehyde and antioxidant activity of wine were evaluated at 0, 15, 30, 45, 60 and 75 days of storage, while monomeric and polymeric phenolics were analyzed at 0, 30 and 75 days of storage. The loss of acetaldehyde was fitted to a first-order reaction model, the rate constant (k) demonstrated that different chemical reaction happened in wines containing a different initial acetaldehyde. The disappearance of monomeric phenolics and the formation of polymeric phenolics induced by acetaldehyde could be divided into two phases, the antioxidant activity of wine did not alter significantly in the first phase, although most monomeric phenolics vanished, but the second phase would dramatically reduce the antioxidant activity of wine. Furthermore, a higher level of acetaldehyde could shorten the reaction time of the first phase. These results indicate that careful vinification handling aiming at controlling the acetaldehyde allows one to maintain prolonged biological activity during wine aging.

## 1. Introduction

Acetaldehyde is the most abundant volatile aldehyde in wines, and it is not only an enzymatically-derived byproduct of wine yeast metabolism during and after alcohol fermentation [1,2,3], but also the major product of wine chemical oxidation [4]. In commercial red wines, a large concentration range of acetaldehyde from below a limit of detection to 211 mg/L was found, which attributed to different vinification management [5]. Actually, acetaldehyde can be responsible for some beneficial reactions, involving mainly phenolics that could improve wine color stability and astringency over time through the formation of phenolic condensation products [6,7,8]. High levels of acetaldehyde produced by yeast-mediated micro-oxygenation exhibited a faster evolution of phenolic compounds during wine bottle aging, which had a similar effect of bottle closures with increased oxygen ingress [9], and polymeric pigment, which was elevated through acetaldehyde-mediated reactions, was also found to be closely related to dry red wine age [9,10]. Thus, acetaldehyde should play a key role during red wine aging.

Polyphenols determine the color, flavor, astringency, bitterness and aging behavior of red wine [11,12], which are all closely relevant to wine quality. Besides, antioxidant activity is the most fascinating property in relation to the health benefits of red wine consumption, and it has been studied by different in vitro and in vivo methods and related to the presence of polyphenols [13,14]; epidemiological studies have also demonstrated the correlation between long-term wine consumption and increased longevity in the population [15]. Red wine polyphenols include not only grape polyphenols, such as anthocyanins, flavonols, flavanonols, pro-anthocyanidins, phenolic acids and stilbenes, but also new polymeric phenolic products generated during the wine-making process, especially a period of aging in the bottle required by high-quality red wines. Thus, the evaluation of the antioxidant activity and polyphenol levels of red wines has been sought.

As the complexity of polyphenols constituted in red wines, there were opposite conclusions about the biological activity of red wine during aging. The research of red wines obtained from the same commercial wineries but with different vintages demonstrated that the antiradical efficiency of the samples increased during aging [16]; however, young red wines were also found to have higher antioxidant activity than aged wines [17]. The different initial phenolic composition among wines may explain these inconsistent results, as the comparative of antioxidant abilities were not monitored during the aging of the same wine, and all parameters affecting phenolic composition would certainly influence the oxygen consumption effect, as previously observed by other authors [18,19]. Recently, the research of eighty-seven red wines demonstrated that antioxidant activity decreased during 120 months of aging, which was determined by the 1,1-diphenyl-2-picrylhydrazyl (DPPH) radical scavenging abilities [20].

A wide variety of phenolic compounds contribute to the antioxidant ability of wine, resulting in the difficulty of clarifying the relative contribution of each antioxidant species. The antioxidant activity test of different red wine samples demonstrated a relatively high correlation with total flavanols but relatively low correlation with the results from total phenolics and total anthocyanins concentration; this suggested that the flavanols in wine are strong contributors to the antioxidant ability of wine [21]. A study of the antioxidant capacity of oak wood used in wine aging demonstrated that ellagitannins were the compounds mainly responsible for the antioxidant capacity of oak wood, and some phenolic acids were also significantly correlated with antioxidant capacity [22], while anthocyanins were confirmed to be the main compounds with significant contribution to wine bioactivity [23,24].

Even though the chemistry reaction of phenolics in wine mediated by acetaldehyde has been investigated, a comprehensive and systematic study of the correlation between the antioxidant ability of wine and phenolic evolution induced by the acetaldehyde-mediated condensation has not been deciphered yet. The aim of this study is to fully reveal the reaction kinetics of acetaldehyde, and the corresponding changes in phenolic composition and antioxidant activity during the aging of Merlot wine containing different levels of acetaldehyde. Additionally, the outcome of such a study was expected to provide further insights into the evolution of antioxidant activity during wine aging, and advance the knowledge on the proper management of acetaldehyde accumulation during vinification.

## 2. Results and Discussion

### 2.1. Antioxidant Activity during Treatment

**Figure 1:**
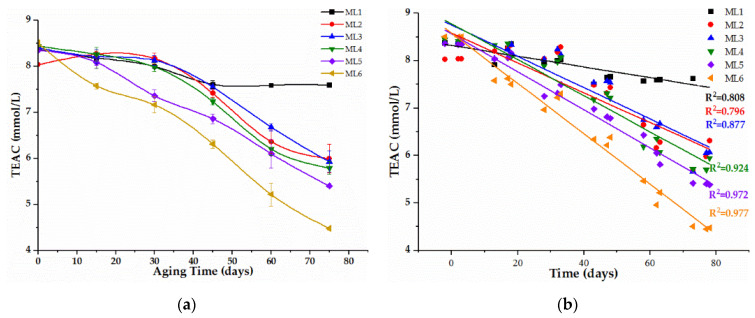
Evolution of antioxidant activities (**a**) and its correlation with aging time (**b**) during wine aging of 75 days. Note: (**a**), each spot represents a set of the arithmetic average ± standard deviation of one measurement, each performed on triplicate bottles; (**b**), each spot represents a set of data of each measurement performed on each bottle and font color for “R^2^” is consistent with color for each correspondent spot. Abbreviations: TEAC, antioxidant activities were expressed as Trolox equivalents per liter of wine (mM TEAC/L); ML1, wines containing 7.86 ± 0.10 mg/L acetaldehyde; ML2, wines containing 41.72 ± 1.97 mg/L acetaldehyde; ML3, wines containing 61.19 ± 3.93 mg/L acetaldehyde; ML4, wines containing 128.10 ± 3.08 mg/L acetaldehyde; ML5, wines containing 203.02 ± 6.00 mg/L acetaldehyde; ML6, wines containing 259.02 ± 4.95 mg/L acetaldehyde.

The effects of the different aging time on the antioxidant activities of samples containing different initial acetaldehyde levels were evaluated by the DPPH assay, which has been proven to be a simple and efficient method for the evaluation of the antiradical activity of red wine [25,26]. The results were expressed as Trolox equivalents per liter of wine (mM TEAC/L) and were shown in Figure 1a and Table S2 (original data). No obvious correlation (R^2^ = 0.27) was found between the antioxidant activities of wines and initial acetaldehyde levels when samples were analyzed before aging (0 day); thus, the interference of acetaldehyde on DPPH assays could be eliminated. The DPPH assays of all the wines gave the observably declining trends along with aging except for ML1, which was without the acetaldehyde addition, and no increasing point was found during 75 days of aging, indicating that acetaldehyde could induce the reduction of the total antioxidant activities of red wines, although the ethyl-linked anthocyanin-flavanol pigments mediated by acetaldehyde demonstrated stronger antioxidant activities than their precursor anthocyanins in the research of model wine [27].

Moreover, different aging time had different responses of antioxidant activities with the participation of different levels of acetaldehyde. After 15 days of wine aging, only ML6 containing the highest acetaldehyde had the dramatically reduction of antioxidant activities, while other wines had stable antioxidant activities. Otherwise, ML5 also exhibited the significant reduction of antioxidant activities after 30 days of wine aging compared with the initial wines, and antioxidant activities of all the wines containing exogenous acetaldehyde generally had been distinctly declining for 45 days. Furthermore, ML6 had the constant largest reduction rate of antioxidant activities during the 75 days, followed by ML5, which also had the invariable reduction rate except for the first 15 days. These results explained that differences in variation found in antioxidant activities among samples during aging were probably due to the distinguishing phenolic reaction mediated by different levels of acetaldehyde; the really high levels of acetaldehyde (above 250 mg/L) in ML6 altered the conventional evolution of phenolics, which was the major contributor of antioxidant activities in wine.

The correlations between antioxidant activity of all wines (ML1, ML2, ML3, ML4, ML5 and ML6) and aging time was shown in Figure 1b. Stronger negative correlations were generally observed when wine contained the higher initial level of acetaldehyde. Thus, these variational correlations reflected a dominant position of chemical reaction induced by acetaldehyde during the evolution of antioxidant activities. When acetaldehyde concentration was above 200 mg/L (ML5), R^2^ did not increase sharply, but remained stable at 0.972 and 0.977, respectively for the ML5 (above 200 mg/L) and ML6 (above 250 mg/L).

### 2.2. Acetaldehyde and Polymeric Phenolics during Treatment

**Figure 2:**
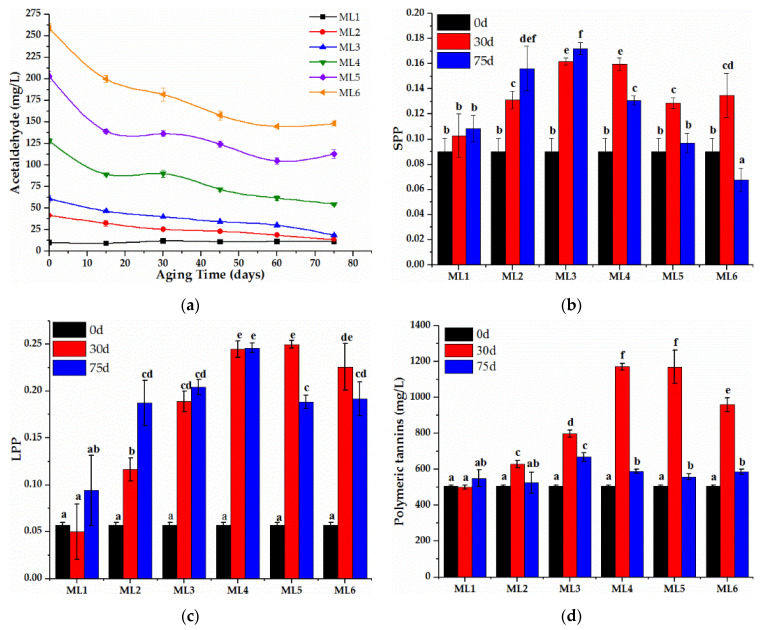
Evolution of acetaldehyde (**a**), SPP (**b**), LPP (**c**) and PT (**d**) during wine aging of 75 days. Different letters indicate significant differences at the 0.05 level. Abbreviations: ML1, wines containing 7.86 ± 0.10 mg/L acetaldehyde; ML2, wines containing 41.72 ± 1.97 mg/L acetaldehyde; ML3, wines containing 61.19 ± 3.93 mg/L acetaldehyde; ML4, wines containing 128.10 ± 3.08 mg/L acetaldehyde; ML5, wines containing 203.02 ± 6.00 mg/L acetaldehyde; ML6, wines containing 259.02 ± 4.95 mg/L acetaldehyde; SPP, short polymeric pigments; LPP, large Polymeric Pigments; PT, polymeric tannins.

Variations in the concentrations of acetaldehyde present during the aging of the Merlot wines were shown in Figure 2a and Table S3 (original data). In the ML1 (CK) wine, the initial acetaldehyde concentration was around 10 mg/L, and there was no systematic change during the aging of 75 days. Acetaldehyde can always be detected in bottle wines after the aging of 10 or 60 years [28], and the remainder of the detection of acetaldehyde in wines was assumed to control wine chemical equilibrium, generally leaving a small amount present in the wine without disappearing and obvious fluctuation when wines are aging under stable circumstances. Although acetaldehyde decreased continuously in all the wines except ML1, no wine had the lower level of acetaldehyde than ML1 at the end of the aging, even for ML2 with the lowest acetaldehyde addition.

Some similar patterns of acetaldehyde evolution could be observed in all Merlot wines containing exogenous acetaldehyde; the first 15 days had the swiftest loss of acetaldehyde and then it flattened out. This trend was consistent with the reaction kinetics of the acetaldehyde-mediated condensation between (−)-epicatechin and anthocyanins in model wine solutions [29]; the evolution curve of formation of ethyl-linked products could be divided into two parts, the initial phase where preliminary polymerization was occurring with a high reaction rate, and the final phase, where the loss of the polymeric pigments predominated with a low reaction rate. The evolution of SPP and LPP fitly verified the reaction regularity of acetaldehyde in wine, as shown in Figure 2b,c (Table S4, original data), which were for SPP and LPP, respectively. Both SPP and LPP increased observably in all wines containing exogenous acetaldehyde during the first 30 days’ of aging (first phase) along with a high reduction rate of acetaldehyde, as shown in Figure 2a, as acetaldehyde gives numerous reactions with free native anthocyanins producing new polymeric pigments [9], while both SPP and LPP also decreased markedly during subsequent aging (second phase) in the ML5 and ML6 wines that also contained considerable levels of acetaldehyde after 30 days of aging; this phase should involve the loss of the polymeric pigments, which may result in the precipitation of pigments under the action of acetaldehyde. However, both SPP and LPP of ML2 continued to increase distinctly during the second phase with the lower level of acetaldehyde, while there was no magnitude for ML1, suggesting that there was the immoderate polymerization induced by acetaldehyde in ML5 and ML6. The increase in PT during the first phase detected only for wines containing exogenous acetaldehyde (Figure 2d and Table S4) suggested that reactions involving acetaldehyde, such as the formation of ethyl bridged flavan-3-ols, probably occurred, which agreed with the previous study [9,30]. However, contrary to expectations, some of the accumulation of PT during the first phase disappeared during the second phase again, which will be needed to be further studied systematically. Furthermore, the copigmentation process in the first step of the new pigments’ formation could enhance flavylium stabilization [31], which should slow the reduction of acetaldehyde during the subsequent aging. Besides, the new formed pigments also may slow down the reaction induced by acetaldehyde, as the present of procyanidin B2 in model wine containing acetaldehyde slowed the loss of the anthocyanins [32].

The reaction kinetic model of acetaldehyde was studied to better understand the chemical reaction during aging. The most regular kinetic models cover zero-order [C_t_ = C_0_ + kt], first-order [C_t_ = C_0_ exp(−kt)], or second-order [1/C_t_ = 1/C_0_ + kt] reaction models, where t is the reaction time (day), k is the rate constant and C_t_ is the compound content at time t, and C_0_ represents the initial compound content. As the previous studies confirmed that the degradation of anthocyanins induced by acetaldehyde or wine aging followed a first-order reaction model [25,29] and anthocyanins were the major phenolics in dry red wines, the reaction rate constants (k) were calculated by using linear regression to plot the natural log (ln) of the ratio of the acetaldehyde remaining at the different reaction time in the wines with different initial acetaldehyde, and the half-life (t_½_) [−ln (0.5) × k^−1^] were also summarized in Table 1. In all cases, the R^2^ values were higher than 0.92, indicating a good data fit to the first-order kinetic model. A general decrease in the values of k, accompanied by a parallel increase in the corresponding values of t_½_, was observed when initial acetaldehyde concentration increased. The diverse values found for t_½_ in wines with different initial acetaldehyde clearly suggested that the aging reaction mediated by acetaldehyde were different; the similar k and t_½_ of ML2 and ML3 confirmed that the analogous reaction happened in these two wines, which were accompanied by the continuous increase in LPP. Conversely, ML5 and ML6 underwent a polymerized further reaction, which resulted in the lower LPP.

The preliminary polymerization (15 days) mediated by acetaldehyde did not bring the distinct variation of the antioxidant activity of wines except ML6, as shown in Figure 1, suggesting that the primitive polymerizate could compensate for the losing antioxidant activity resulting from the reactive and disappeared monomeric phenolics (which will be discussed below), but cannot reinforce the total antioxidant activity of wine, although the ethyl-linked anthocyanin-flavanol pigments mediated by acetaldehyde demonstrated stronger antioxidant activities than their precursor anthocyanins in the research of model wine [27]. The enormous contribution of the concomitant formation of polymerized compounds to antioxidant activity was also found in a previous study; however, the antioxidant activity of red wines decreased only over the first 20 days of aging, and following that, a long period of stabilization up to 120 days was demonstrated [25], which was at opposite poles of the results induced by acetaldehyde in this study (Figure 1). The present substantial acetaldehyde should greatly accelerate the progressive structural modification of the phenolic compounds, which skipped over the reduction phase of antioxidant activity. Furthermore, no distinct difference of antioxidant activity was found during the first 15 days of aging, suggested that the ethyl-linked polymerizate may have higher antioxidant activity than the direct adducts. Considering the fact that antioxidant activity (DPPH assay) during the aging of Cabernet Sauvignon demonstrated a significant increase compared to their respective young wines [33], and the decrease in antioxidant activity over time during aging also was inversely affirmed [25]; the conflicting evolution of the antioxidant activity during different wine aging was likely due to the initial level of acetaldehyde in red wines. However, the further polymerization generated from acetaldehyde would visibly weaken antioxidant activity of wines, attributing to the precipitation of macromolecular polymer or its weak antioxidant activity. Nevertheless, sufficient acetaldehyde in ML6 could also destroy the protective effect from copigmentation or the newly formed pigments, as their antioxidant activity drastically decreased at the first 15 days, indicating that the macromolecular polymer were formed far ahead, which was absolutely different from other wines.

### 2.3. Monomeric Phenolics during Treatment

**Figure 3:**
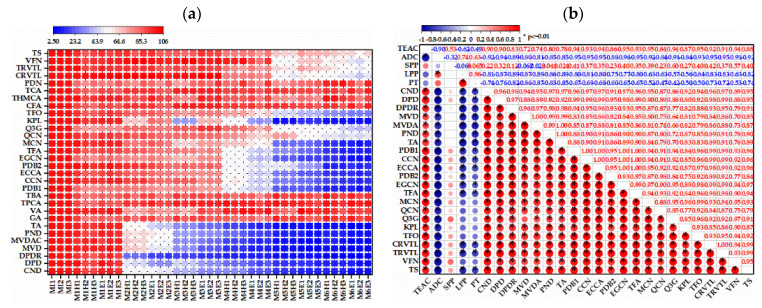
The percentage of initial concentration of each phenolics at different time points during wine aging (**a**) and the correlation of antioxidant activities and key phenolic compounds (**b**). For (**b**), red and blue colors represent positive and negative correlation, respectively; the large and small circular sizes correspond to strong and weak correlations, respectively; * denote statistical significance by Pearson’s correlation analysis at 0.01. Abbreviations: MI1, MI2 and MI3, three bottles of initial wine without exogenous acetaldehyde at 0 days; MiE1, MiE2 and MiE3 (i = 1, 2, 3, 4, 5, 6), three bottles of wine with different initial acetaldehyde (1–6) at 30 days of aging; MiH1, MiH2 and MiH3 (i = 1, 2, 3, 4, 5, 6), three bottles of wine with different initial acetaldehyde (1–6) at 75 days of aging; TEAC, antioxidant activities were expressed as Trolox equivalents per liter of wine (mM TEAC/L); ADC, acetaldehyde consumption; TA, total anthocyanins; TFA, total flavanols; TFO, total flavonols; SPP, short polymeric pigments; LPP, large polymeric pigments; PT, polymeric tannins; CND, cyanidin-3-glucoside; DPD, delphinidin-3-glucoside; DPDR, delphindin 3-O rutinoside; MVD, malvidin-3-glucoside; MVDAC, malvidin-3-glucoside acetate; PND, peonidin-3-glucoside; GA, gallic acid; VA, vanillic acid; TPCA, trans-p-coumaric acid; TBA, total benzoic acid; PDB1, procyanidin b1; CCN, catechin; ECCA, epicatechin; PDB2, procyanidin b2; EGCN, epigallocatechin; MCN, myricetin; QCN, quercetin; KPL, kaempferol; CFA, caffeic acid; THMCA, trans-ferulic acid; TCA, total hydroxycinnamic acid; PDN, polydatin; CRVTL, cis-resveratrol; TRVTL, trans-resveratrol; VFN, viniferin; TS, total stilbenes.

The variation of each monomeric phenolics was determined at 0 (I), 30 (H) and 75 (E) days of wine aging, including anthocyanins, hydroxybenzoic acids, flavanol, flavonol, hydroxycinnamic acids and stilbenes (Table S5, original data). Figure 3a showed the percentage of the initial concentration of each phenolics at different time points (15 and 30 days) for wine samples with different acetaldehyde addition (1–6). According to Figure 3a, anthocyanins were the most sensitive group responding positively to the acetaldehyde level, more than 50% of the compounds vanished when the initial acetaldehyde was 60 mg/L after 30 days of aging for M3H wine, followed by flavanols, which all decreased 50% when the initial acetaldehyde was 60 mg/L after 75 days of aging for M3E, then there were the flavonols for M4E containing 130 mg/L initial acetaldehyde after 75 days of aging. There were fluctuations in the evolution of hydroxybenzoic acids and hydroxycinnamic acids in various aging periods of wines with different initial acetaldehyde, indicating that these compounds did not become very involved in the aging reaction induced by acetaldehyde. This order of reaction induced by acetaldehyde agreed with the loss of some phenolics promoted by acetaldehyde after a wine bottle aging of one year, in which monomeric anthocyanins had the largest decrease, followed by the flavonols, flavonoids and benzoic acids, with the hydroxycinnamic acids having the smallest percentage loss [9].

The large decrease in cis-resveratrol and viniferin were found in ML6 wine with very high levels of acetaldehyde (above 260 mg/L); this means that these compounds may participate in extreme polymerization reaction mediated by acetaldehyde. There is not much literature data concerning the evolution profile of stilbenes during wine aging. In general, the evolution of resveratrol during aging in the bottle did not follow defined trends, and the stilbenes as a group were not influenced significantly by the time factor [34]. Thus, one point that deserves further research is that the potential footprint of the remaining metastable monomeric and newly formed polymerized phenolics in red wines after long-time aging when reactive phenolics, including monomeric anthocyanins, flavanol and flavonol, has disappeared. After all, very noticeable antioxidants of wines were existing (Figure 1), while most of the reactive monomeric phenolics vanished with the action of acetaldehyde after 30 days of aging.

Because the qualitative change of reactive phenolics was found in wines containing exogenous acetaldehyde after an aging of 30 days, the comparative analysis of Pearson correlations between acetaldehyde consumption and reactive phenolics (anthocyanins, flavanols and flavonols) in the wines at this point were shown in Figure 3b, which were all above 0.8; the results were consistent with above findings (Figure 3a). The Pearson correlations between antioxidant activities and different types of phenols of wines after the aging of 30 days were also investigated, as shown in Figure 3b. As expected, anthocyanins, flavanol and flavonol, which can easily react with acetaldehyde, were also positively and highly correlated with the antioxidant parameters, and the correlation of these phenols with TEAC all reached highly significant levels (*p* < 0.01) in all wines. Comparing classes of reactants, the order of reactivity with acetaldehyde in wine was monomeric anthocyanins, flavanols and flavonols, but the order of the *r* between these flavonoid compounds and the antioxidant capacity values of red wines measured by DPPH was flavanols (0.86–0.95), flavonols (0.84–0.95) and anthocyanins (0.72–0.90). According to the previous study, the authors determined that the ortho 3,4-dihydroxy moiety in the B ring (e.g., in catechin and quercetin), the meta 5,7-dihydroxy arrangements in the A ring (e.g., in kaempferol), the 2,3-double bond in combination with both the 4-keto group and the 3-hydroxyl group in the C ring (e.g., in quercetin) and the o-dihydroxy structure in the B ring impart greatest antioxidant activity [35]. Thus, flavonols (quercetin, myricetin and kaempferol) had higher antioxidant activity compared with flavan-3-ols (catechin) resulting from the 2,3-double bond in the C-ring and 4-oxo function [26], which was at a variance with the results from the wines containing the exogenous acetaldehyde.

Red wine constitutes a dynamic system during fermentation and aging, resulting in the continuous variation of active constituents involving numerous monomeric, polymerized and condensed phenolic compounds that undoubtedly affect its antioxidant effect. Although SPP presented a positive correlation with the antioxidant parameters (0.53), as the potential major antioxidant substance, polymeric phenolics (LPP and PT) were negatively correlated with the antioxidant parameters, as shown in Figure 3b. Thus, it is really hard to assess the antioxidant activity of each phenolics in red wines according to their evolution. A further understanding of the antioxidant activity of polymeric phenolics needs to be elucidated systematically in the future, while separation and purification of the phenolic compound-derived polymers and the more precise analytical approach will be a challenge.

According to the Pearson correlations between polymeric phenolics (SPP, LPP and PT) and different types of phenols after the aging of 30 days (Figure 3b), we could suggest that the formation of LPP should depend primarily on monomeric anthocyanins (0.81–0.89), then, flavanols (0.75–0.81) and flavonols (0.56–0.64); the similar results were obtained for the formation of PT. However, different conditions were found in the formation of SPP; the primary monomeric phenolics were flavonols (0.23–0.60), then were flavanols (0.23–0.41) and anthocyanins (0.00–0.32). These results could be helpful in understanding phenolic evolution during wine aging.

**Figure 4:**
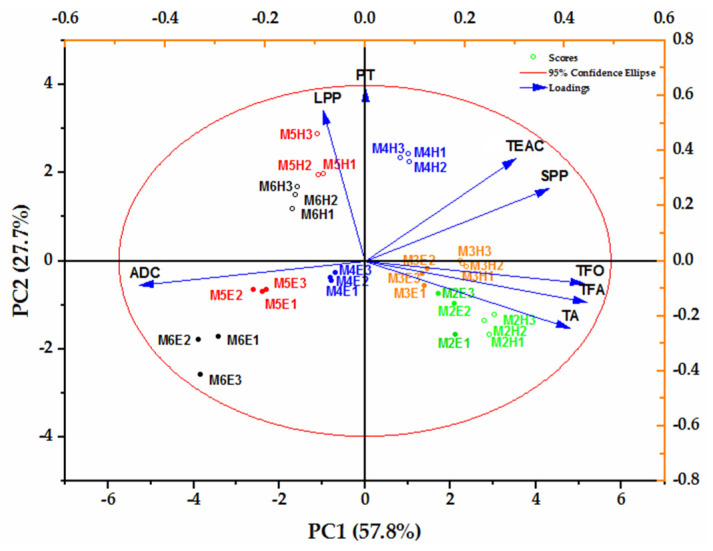
Principal component analysis of acetaldehyde, TEAC and key phenolic compounds data at 30 and 75 days of wine aging. Abbreviations: MiE1, MiE2 and MiE3 (i = 2, 3, 4, 5, 6), three bottles of wine with different initial acetaldehyde (2–6) at 30 days of aging; MiH1, MiH2 and MiH3 (i = 2, 3, 4, 5, 6), three bottles of wine with different initial acetaldehyde (2–6) at 75 days of aging; TEAC, antioxidant activities expressed as Trolox equivalents per liter of wine (mM TEAC/L); ADC, acetaldehyde consumption; TA, total anthocyanins; TFA, total flavanols; TFO, total flavonols; SPP, short polymeric pigments; LPP, large polymeric pigments; PT, polymeric tannins.

As discussed above, anthocyanins, flavanols and flavonols were phenolic compounds with the highest positive contribution to the DPPH value, while polymeric phenolics were the ones with the highest potential contribution. Furthermore, these compounds were all closely related to the aging reaction induced by acetaldehyde. Figure 4 showed the biplot graphic, where it represented the association of these key polyphenols (groups) with the DPPH value and acetaldehyde consumption in the ML wines studied. As could be observed in Figure 4, the content of SPP, TFO, TFA and TA were higher in wines with lower levels of acetaldehyde, while LPP demonstrated the higher content in wines with a higher level of acetaldehyde (PC1, 57.8%). These intuitive results can explain the evolution of the bioactivity of wines mediated by acetaldehyde. Differences were also observed in the evolution of wines that contained different initial acetaldehyde. As was shown in Figure 4, a greater difference was found between the response of the aging of 30 and 75 days when the wines contained higher initial acetaldehyde (PC2, 27.7%). No difference could be observed in the wines with the first two levels of acetaldehyde (ML2 and ML3), but from the ML4 wine, a clear difference appeared, which affected all the distinct phenolic profiles. These results were in accordance with to our previous study, where we reported acetaldehyde levels at bottling as the leading cause of the phenolic compounds’ profile evolution during wine aging [9]. Regarding this result, the aging potential of dry red wine should be predicted in advance according to the response of various degrees of reactions induced by acetaldehyde, which would be an artificial simulated wine-aging technology.

## 3. Materials and Methods

### 3.1. Reagents and Standards

The phenolic standards including malvidin-3-*O*-glucoside, procyanidin dimers B1, gallic acid, (+)catechin, (−)epicatechin, procyanidin dimers B2, (−)-epigallocatechin, caffeic acid, myricetin, quercetin, quercetin 3-glucoside, vanillic acid, trans-p-coumaric acid, 3,4-dihydroxybenzoic acid, polydatin, kaemperol, cis-resveratrol, trans-resveratrol, trans-4-hydroxy-3-methoxycinnamic acid and viniferin were purchased from Sigma-Aldrich (St. Louis, MO, USA). The water was purified using a Milli-Q system (Millipore, Billerica, MA, USA). Ortho-phosphoric acid was obtained from Tianjin kemio chemical reagent Co., Ltd. (Tianjin, China). Acetonitrile was obtained from Fisher Scientific (Fair Lawn, NJ, USA). Acetaldehyde, DNPH-acetaldehyde hydrazine standard, DNPH (30% water, *m/m*) and bovine serum albumin were obtained from Sigma-Aldrich (St. Louis, MO, USA), while the DNPH (30% water, *m/m*) was purified by recrystallization from acetonitrile. All above solvents employed were high-performance liquid chromatography (HPLC grade). DPPH was purchased from Sigma-Aldrich (St. Louis, MO, USA).

### 3.2. Wines

One dry red wine sample, 100% *Vitis. vinifera* L. cv. Merlot, was obtained from the Chateau Changyu Baron Balboa winery, Xinjiang, China. The wine fermentation was performed on healthy grape berries, the Merlot grape with 25.1 °B during the 2017 harvest season, which was prepared with standard winemaking techniques using the *Saccharomyces cerevisiae* yeast Lalvin^®^ RC212 (Lallemand, St. Simom, France). After the completion of spontaneous malolactic fermentation (MLF) and sterile filtration (without extra SO_2_ addition), the wine was separated into six lots of 6 L using a 10 L sterile glass jar with N_2_ protection, then acetaldehyde was added to the wines so as to have six wines with six different acetaldehyde levels: ML1 (7.86 ± 0.10 mg/L acetaldehyde, CK), ML2 (41.72 ± 1.97 mg/L acetaldehyde), ML3 (61.19 ± 3.93 mg/L acetaldehyde), ML4 (128.10 ± 3.08 mg/L acetaldehyde), ML5 (203.02 ± 6.00 mg/L acetaldehyde) and ML6 (259.02 ± 4.95 mg/L acetaldehyde). The high acetaldehyde level of ML6 referred to the research of the exogenous acetaldehyde during vinification [8]. These six lots were then bottled in 375 mL clear-glass bottles with N_2_ protection, and the bottles were closed with the same type closure and volumetric headspace. These wines were stored at room temperature (25–28 °C) for 75 days. For this study, three bottles of each wine were analyzed respectively after 15, 30, 45, 60 and 75 days of aging.

The base parameters (mean ± standard deviation) of the Merlot wine before the acetaldehyde addition were: ethanol content 14.32 ± 0.13% *v*/*v*, pH 3.73 ± 0.01, titratable acidity 5.41 ± 0.08 g/L ex-pressed as tartaric acid, residual sugars 0.40 ± 0.02 g/L and volatile acidity 0.50 ± 0.02 g/L.

### 3.3. Antioxidant Capacity Assays

The total antioxidant activity of wine was evaluated according to radical scavenging in vitro with DPPH antiradical assays [36]. The DPPH radical solution (1 mM) was prepared daily in ethanol and diluted to an absorbance of 0.900 ± 0.05, which was also protected from light. The absorbance was recorded to check the stability of the radical throughout the time of analysis. Each sample with a fixed volume of 0.1 mL was mixed with 0.9 mL DPPH solution stored in the dark at room temperature and determined at time t = 0 min and 30 min at 517 nm using an Agilent Cary 60 UV-Vis spectrophotometer (Agilent Technologies, Santa Clara, CA, USA). The analytical standard (Trolox) was used to construct the calibration curves (0.2–2.0 mM/L); the DPPH radical scavenging activity was expressed as Trolox equivalents per liter of wine (mM TEAC/L).

### 3.4. Analysis of Acetaldehyde

A 2,4-dinitrophenylhy-drazine (DNPH) derivatization–HPLC method was used to analyze acetaldehyde in the wine [28]. A total of 100 μL of wine was dispensed to a 2 mL vial, followed by 20 μL of freshly prepared 1120 mg/L SO_2_ solution, then 20 μL of 25% sulfuric acid (*v*/*v*) was added, followed by 140 μL of 8 g/L DNPH reagent. The solution was allowed to react for 15 min at 65 °C and then promptly cooled to room temperature. A completely derivatized reaction solution was analyzed by Waters e2695 HPLC (Milford, MA, USA) equipped with a 2998 diode array detector after it was filtered by 0.20 μm MicroLiter PTFE membrane filters (Jinteng Experimental Equipment Co., Ltd., Tianjin, China). Separation occurred on An Agilent ZORBAX Rapid Resolution HT, SB-C18 column (1.8 μm, 4.6 × 100 mm) held at 35 °C with a flow rate of 0.75 mL/min. The analyses were quantified at 365 nm using external calibration standards with a linear regression analysis. The identification of the acetaldehyde-DNPH was made by comparison with retention times and the chromatographic profile reported by Han et al. [28]. The concentrations of acetaldehyde were computed using an external standard method. Data analysis and peak integration was carried out using the Empower 3 chromatography workstation.

### 3.5. Analysis of Polymeric Tannins

The HPLC separation and quantification of polymeric tannins (PT) was analyzed using Waters e2695 HPLC (Milford, MA, USA) equipped with a 2998 diode array detector after the wine sample was filtered by 0.20 μm MicroLiter PTFE membrane filters (Jinteng Experimental Equipment Co., Ltd., Tianjin, China), which was according to the Peng et al. method [37]. An Agilent PLRP-S 100-Å reversed-phase polystyrene divinyl benzene column (4.6 × 150 mm, 3 μm particle size) protected with a guard cartridge with the same packing material (PLRP-S, 5 × 3 mm) and kept at 35 °C was selected. The elution solvents were: solvent A, 1.5% *v*/*v* ortho-phosphoric acid (Tianjin kemio chemical reagent Co., Ltd., Tianjin, China), and solvent B, consisting of 80% acetonitrile (HPLC grade, Fisher Scientific, Fair Lawn, NJ, USA)) with 20% of solvent A. The following gradient was selected: 0time conditions, B 6%; 73 min, B 31%; 78 min, B 62%, staying constant until 86 min; 90 min, B 6%. The flow rate of the mobile phase was 1 mL/min, and 20 µL of wine or calibration standards were injected onto the column. The detection was performed by monitoring the absorbance signals at 280 nm and quantified at as mg/L of (+)-catechin (Sigma, St. Louis, MO, USA). Data analysis and peak integration was carried out using the Empower 3 chromatography workstation.

### 3.6. Analysis of Short Polymeric Pigments and Large Polymeric Pigments

Short polymeric pigments (SPP) and large polymeric pigments (LPP) were determined by the Harbertson–Adams assay [38]. Briefly, the first tube was made up by dispensing 1 mL of the acetic acid/NaCl buffer into the tube and then adding 500 µL of the wine sample, then 80 µL of 0.36M potassium metabisulfite was added, mixed, and the absorbance at 520 nm was determined again after a 10 min incubation (reading A). For the second tube, 1 mL of the acetic acid/NaCl buffer containing bovine serum albumin (1 mg/mL) was put into a microfuge tube, and 500 µL of the wine sample was added. The mixture was allowed to incubate at room temperature for 15 min with slow agitation. After incubation, the sample was centrifuged for 5 min at 135,000 g to pellet the precipitate. One milliliter of the supernatant was put into a cuvette, then 80 µL of 0.36 M potassium metabisulfite was added, and after a 10-min incubation the absorbance was determined at 520 nm (reading B). The absorbance due to SPP and LPP is given as A and (A-B), respectively.

### 3.7. Analysis of Monomeric Phenolics

Anthocyanins and non-anthocyanin phenolics were analyzed as previously described [39,40]. Briefly, the monomeric anthocyanins were detected by an HPLC (LC-20A; Shimadzu, Kyoto, Japan) at 535 nm, and a C18 chromatographic column (4.6 × 250 mm; 5 μm; Atlantis C18; Waters, Saint-Quentin-en-Yvelines, France) was used. The conditions of analysis were as follows: column temperature, 30 °C; phase A, water/acetonitrile/formic acid 80:10:7 *v*/*v*/*v*; and phase B, water/acetonitrile/formic acid 40:50:7 *v*/*v*/*v*; flow rate, 1.0 mL/min; injection amount, 20 µL. Mobile phase (A) comprised formic acid/water/acetonitrile (5:40:55, *v*/*v*/*v*), and mobile phase (B) comprised formic acid/water/acetonitrile (10:40:50, *v*/*v*/*v*). The gradient elution protocol (*v*/*v*) had the following composition: 0.00−15.00 min, 0−10% B; 15.00−30.00 min, 10−20% B; 30.00−45.00 min, 20%−35% B; 45.00−46.00 min, 35−100% B; 50.00−51.00 min, 100−0% B. The relative concentration of each anthocyanin was expressed as the malvidin-3-O-glucoside equivalent.

The individual non-anthocyanin phenolics were analyzed by a UPLC−MS/MS (ultrahigh-performance liquid chromatography−tandem mass spectrometry, NEXERA LC-30AD; Shimadzu, Kyoto, Japan) coupled to a triple quadrupole mass spectrometer (AB Sciex QTRAP 4500 LC/MS/MS system; AB Sciex, Redwood City, CA, USA). Samples were eluted using a C18 chromatographic column (1.7 μm; 2.1 × 100 mm; ACQUITY UPLC BEH; Waters, Saint-Quentin-en-Yvelines, France) and Mobile phase comprised of (A) water/formic acid (999:1, *v*/*v*) and (B) acetonitrile/formic acid (999:1, *v*/*v*). The gradient elution protocol (*v*/*v*) had the following composition: 0.00−3.00 min, 5% B; 3.00−6.00 min, 30% B; 6.00−9.00 min, 50% B; 9.00−12.00 min, 70% B; 12.00−17.00 min, 5% B. For MS conditions, the negative ion mode was used with the following conditions: multiple reaction monitoring detection (transitions information in Table S1), 500 °C ion source temperature, 40 psi curtain gas, 60 psi nebulizer gas, medium collision gas, 50 psi auxiliary heat gas and −4500 V ion spray voltage. Each non-anthocyanin phenolic was identified by retention time with standards and weight of molecular ion and fragment ion. The concentrations of the non-anthocyanin phenolics were computed using an external standard method.

### 3.8. Statistical Analysis

Data were expressed as the mean of one measurement, each performed on triplicate bottles. Correlation and regression analysis and one-way analysis of variance (ANOVA) were performed using the IBM SPSS Statistics 25 (IBM Corp., Armonk, NY, USA). Differences among polymeric phenolics (Figure 2) or the reaction rate constants (Table 1) were determined by the Duncan’s test. The principal component analysis (PCA) was performed using Origin 2022 software with the PCA-app (OriginLab Corp., Northampton, MA, USA) to obtain biplot graphics (Figure 4).

## 4. Conclusions

The reaction kinetics of acetaldehyde and the consequent variation of the phenolic profile and antioxidant ability of dry red wines were investigated in wines containing different initial acetaldehydes in this study. The results certified that the loss of acetaldehyde followed the first-order reaction and the rate constant (k) and half-life t_½_ demonstrated that the acetaldehyde-mediated condensation was enhanced by the higher level of acetaldehyde. The primary polymerization products from the monomeric anthocyanins, flavanol and flavonol mediated by acetaldehyde had the considerable antioxidant ability that would make up for a loss of monomeric phenolics to stabilize the total antioxidant ability of wine, but a further polymerization product would have the opposite effect. Future studies could address the antioxidant ability of different extents of the polymerization of phenolics mediated by acetaldehyde. Although acetaldehyde may contribute to the improvement of organoleptic properties in fresh red wines, the proper management of acetaldehyde levels will benefit the prolonged biological activity of red wine.

## Figures and Tables

**Table 1:** Kinetic parameters of the loss of acetaldehyde in Merlot wines during aging reaction.

Wines	IA (mg/L) ^a^	K (days^−1^) ^b^	R^2^	t_½_ (day) ^c^
ML2	41.72 ± 1.97	0.0142 ± 0.000955 A ^d^	0.9895 ± 0.003221	48.85 ± 3.29 A ^d^
ML3	61.19 ± 3.93	0.0140 ± 0.00110 A	0.9805 ± 0.01312	49.73 ± 3.9 A
ML4	128.10 ± 3.08	0.0121 ± 0.000567 B	0.9785 ± 0.003176	57.45 ± 2.77 B
ML5	203.02 ± 6.00	0.00996 ± 0.000630 C	0.9276 ± 0.01137	69.76 ± 4.3 C
ML6	259.02 ± 4.95	0.00917 ± 0.000589 C	0.9559 ± 0.009559	75.8 ± 4.69 C

^a^ Initial acetaldehyde concentration. ^b^ First-order reaction rate constants. ^c^ Time required for 50% reduction of the initial concentration of acetaldehyde. ^d^ Mean of three replicates ± standard error; means in the same column with different uppercase letters (A, B and C) were significantly different (Duncan, *p* < 0.05) for the five wines with different initial acetaldehyde during reaction.

## Data Availability

Not applicable.

## Supplementary Materials

The following supporting information can be downloaded at: https://www.mdpi.com/article/10.3390/molecules27092608/s1, Table S1: List of transitions for non-anthocyanins phenolics used in this trial; Table S2: The DPPH assay (mM TEAC/L) during wine aging; Table S3: Acetaldehyde (mg/L) during wine aging; Table S4: Polymeric phenolics during wine aging; Table S5: Monomeric phenolics during wine aging.

## Sample Availability

Samples of the compounds are available from the authors.
