# Supplementary material for: Impact of the Acetaldehyde-Mediated Condensation on the Phenolic Composition and Antioxidant Activity of Vitis vinifera L. Cv. Merlot Wine
Source: Molecules. 2022 Apr 19;27(9):2608. doi: 10.3390/molecules27092608 (PMC9105304; doi:10.3390/molecules27092608)
Supplement: Supplementary file 1 [file molecules-27-02608-s001.zip › molecules-1669782-supplementary.pdf]

Supplementary Data for Impact of the Acetaldehyde-mediated Condensation on the Phenolic Composition and Antioxidant Activity of *Vitis vinifera* L. Cv. Merlot Wine

Lingmin Dai [1], Ke Zhong [1], Yan Ma [1], Xiaoqian Cui [1], Yuhang Sun [1], Ang Zhang [2] and Guomin Han [1,*]

[1] School of Bioengineering, Qilu University of Technology (Shandong Academy of Sciences), Jinan 250353, China; dailingmin@qlu.edu.cn (L.D.); zk17854117992@163.com (K.Z.); 13287791781@163.com (Y.M.); 17863908085@163.com (X.C.); syhwssy@163.com (Y.S.)
[2] Technology Centre of Qinhuangdao Customs, Qinhuangdao 066004, China; zhanganggrape@hotmail.com
* Correspondence: gmhan@qlu.edu.cn

Table S1. List of transitions for non-anthocyanins phenolics used in this trial.

**Non-anthocyanins phenolics**	**[M-H]$^-$ (*m*/*z*)**	**MS^2 ions (*m*/*z*)**
Myricetin	479	317*, 179
Quercetin	463	300*, 271
Kaempferol	447	285*, 100
Catechin	289	245*, 203
Epicatechin	289	245*, 203
Epigallocatechin	457	169*, 125
Procyanidin b1	577	289*, 407
Procyanidin b2	577	289*, 407
Polydatin	389	227*, 185
Viniferin	453	347*, 359
trans-Resveratrol	227	185*, 143
Caffeic acid	179	135*, 134
trans-Ferulic acid	193	134*, 178
trans-p-Coumaric acid	163	119*, 93
Gallic acid	169	125*, 79
Vanillic acid	167	152*, 108

Note: M$^-$: negative charged molecular ion; MS^2: fragmentation of M$^-$; *: quantitative ion.

Table S2. The DPPH assay (mM TEAC/L) during wine aging

Wines	0 day	15 days	30 days	45 days	60 days	75 days
ML1-bottle1	8.38	7.92	7.95	7.51	7.57	7.63
ML1-bottle2	8.36	8.26	8.00	7.64	7.59	7.56
ML1-bottle3	8.37	8.36	8.03	7.66	7.6	7.57
ML2-bottle1	8.02	8.19	8.04	7.49	6.65	5.69
ML2-bottle2	8.03	8.27	8.18	7.31	6.16	5.98
ML2-bottle3	8.03	8.32	8.29	7.43	6.28	6.31
ML3-bottle1	8.39	8.05	8.03	7.54	6.75	5.66
ML3-bottle2	8.40	8.24	8.24	7.57	6.60	6.05
ML3-bottle3	8.40	8.34	8.12	7.55	6.68	6.07
ML4-bottle1	8.44	8.33	7.89	7.18	6.19	5.72
ML4-bottle2	8.41	8.36	7.98	7.31	6.36	5.71
ML4-bottle3	8.43	8.09	8.07	7.21	6.07	5.95
ML5-bottle1	8.36	8.04	7.25	6.98	6.43	5.42
ML5-bottle2	8.34	8.06	7.31	6.82	6.05	5.40
ML5-bottle3	8.35	8.16	7.5	6.79	5.81	5.39
ML6-bottle1	8.50	7.58	6.97	6.34	5.46	4.51
ML6-bottle2	8.50	7.63	7.22	6.21	4.96	4.45
ML6-bottle3	8.50	7.51	7.30	6.38	5.22	4.48

Abbreviations: TEAC, the DPPH radical scavenging activity was expressed as Trolox equivalents per litre of wine. ML1, wines containing 7.86±0.10 mg/L acetaldehyde; ML2, wines containing 41.72±1.97 mg/L acetaldehyde; ML3, wines containing 61.19±3.93 mg/L acetaldehyde; ML4, wines containing 128.10±3.08 mg/L acetaldehyde; ML5, wines containing 203.02±6.00 mg/L acetaldehyde; ML6, wines containing 259.02±4.95 mg/L acetaldehyde.

Table S3. Acetaldehyde (mg/L) during wine aging

Wines	0 day	15 days	30 days	45 days	60 days	75 days
ML1-bottle1	10.38	9.37	12.16	10.75	10.93	10.83
ML1-bottle2	9.89	9.12	11.72	11.56	11.94	11.21
ML1-bottle3	10.06	9.52	11.73	11.10	11.05	11.53
ML2-bottle1	43.34	28.72	26.88	22.87	18.39	13.56
ML2-bottle2	42.28	35.66	24.24	23.63	19.05	13.95
ML2-bottle3	39.53	32.70	24.43	22.79	19.04	13.91
ML3-bottle1	56.79	45.04	39.70	35.18	30.61	17.83
ML3-bottle2	62.45	47.30	40.32	34.12	30.11	20.16
ML3-bottle3	64.34	48.07	40.68	33.78	30.00	18.39
ML4-bottle1	128.91	87.48	94.39	74.22	58.89	52.76
ML4-bottle2	124.69	90.45	86.55	70.44	64.25	55.85
ML4-bottle3	130.69	89.99	89.12	70.91	62.38	55.56
ML5-bottle1	197.10	136.72	138.91	120.50	102.14	110.81
ML5-bottle2	209.10	139.01	137.72	126.59	103.56	109.30
ML5-bottle3	202.87	141.59	132.60	126.10	108.81	118.84
ML6-bottle1	255.83	196.63	189.23	162.43	143.95	147.31
ML6-bottle2	264.72	198.40	174.15	151.70	146.44	146.40
ML6-bottle3	256.51	204.50	182.19	159.32	144.02	151.75

Abbreviations: ML1, wines containing 7.86±0.10 mg/L acetaldehyde; ML2, wines containing 41.72±1.97 mg/L acetaldehyde; ML3, wines containing 61.19±3.93 mg/L acetaldehyde; ML4, wines containing 128.10±3.08 mg/L acetaldehyde; ML5, wines containing 203.02±6.00 mg/L acetaldehyde; ML6, wines containing 259.02±4.95 mg/L acetaldehyde.

Table S4. Polymeric phenolics during wine aging

Wines	Aging time (days)	LPP	SPP	PT (mg/L)
MLI-bottle1	0	0.055	0.078	509.65
MLI-bottle2	0	0.056	0.092	510.05
MLI-bottle3	0	0.06	0.099	496.72
ML1-bottle1	30	0.053	0.111	496.86
ML1-bottle2	30	0.019	0.143	490.32
ML1-bottle3	30	0.078	0.115	512.24
ML1-bottle1	75	0.114	0.105	496.59
ML1-bottle2	75	0.051	0.112	567.73
ML1-bottle3	75	0.118	0.117	585.3
ML2-bottle1	30	0.122	0.124	621.54
ML2-bottle2	30	0.134	0.131	612.86
ML2-bottle3	30	0.131	0.138	653.4
ML2-bottle1	75	0.166	0.158	458.74
ML2-bottle2	75	0.177	0.173	544.88
ML2-bottle3	75	0.198	0.138	572.82
ML3-bottle1	30	0.143	0.161	806.00
ML3-bottle2	30	0.145	0.165	774.87
ML3-bottle3	30	0.15	0.159	811.16
ML3-bottle1	75	0.181	0.169	641.27
ML3-bottle2	75	0.186	0.178	680.63
ML3-bottle3	75	0.184	0.17	684.99
ML4-bottle1	30	0.226	0.161	1176.41
ML4-bottle2	30	0.21	0.164	1188.13
ML4-bottle3	30	0.225	0.154	1151.71
ML4-bottle1	75	0.196	0.127	601.83
ML4-bottle2	75	0.195	0.134	589.20
ML4-bottle3	75	0.204	0.132	576.82
ML5-bottle1	30	0.224	0.124	1097.89
ML5-bottle2	30	0.214	0.13	1138.97
ML5-bottle3	30	0.245	0.132	1273.96
ML5-bottle1	75	0.193	0.093	560.22
ML5-bottle2	75	0.195	0.093	572.85
ML5-bottle3	75	0.19	0.106	541.58
ML6-bottle1	30	0.196	0.122	949.61
ML6-bottle2	30	0.202	0.155	925.94
ML6-bottle3	30	0.207	0.128	1003.22
ML6-bottle1	75	0.142	0.079	598.73
ML6-bottle2	75	0.148	0.062	591.04
ML6-bottle3	75	0.103	0.062	570.31

Abbreviations: ML1, wines containing 7.86±0.10 mg/L acetaldehyde; ML2, wines containing 41.72±1.97 mg/L acetaldehyde; ML3, wines containing 61.19±3.93 mg/L acetaldehyde; ML4, wines containing 128.10±3.08 mg/L acetaldehyde; ML5, wines containing 203.02±6.00 mg/L acetaldehyde; ML6, wines containing 259.02±4.95 mg/L acetaldehyde; SPP, Short polymeric pigments; LPP, Large Polymeric Pigments; PT, polymeric Tannins.

Table S5. Monomeric phenolics during wine aging

Wines	Aging time (days)	CND	DPD	DPDR	MVD	MVDGC	PND	TA	GA	VA	TPCA	TBA	PDB1	CCN	ECCA	PDB2	EGCN	TFA	MCN	QCN	Q3G	KPL	TFO	CFA	THMCA	TCA	PDN	CRVTL	TRVTL	VFN	TS
MLI-bottle1	0	7.51	45.65	32.6	96	69.31	11.08	262.15	60.35	2.62	5.55	68.52	53.98	47.88	39.03	38.89	2.82	182.59	13.78	9.5	6.4	0.43	30.11	23.88	0.62	24.49	1	0.35	2.81	0.1	4.26
MLI-bottle2	0	7.38	46.83	31.06	93.37	69.7	10.89	259.24	62.44	2.68	5.57	70.69	53.05	46.07	38.36	37.36	2.77	177.62	13.58	9.34	6.29	0.42	29.63	23.47	0.62	24.09	0.99	0.35	2.87	0.1	4.31
MLI-bottle3	0	7.44	47.22	32.32	94.14	68.71	10.98	260.82	60.87	2.7	5.51	69.08	53.49	46.45	38.68	38.61	2.79	180.03	13.68	9.41	6.35	0.42	29.86	23.66	0.61	24.28	0.89	0.35	2.89	0.09	4.22
ML1-bottle1	30	7.28	46.22	30.66	93.1	64.6	10.74	252.61	54.59	2.55	5.1	62.24	44.29	46.66	36.87	38.93	2.38	169.14	14.01	9.03	5.06	0.43	28.52	21.84	0.62	22.46	0.83	0.35	2.8	0.1	4.07
ML1-bottle2	30	6.69	42.43	28.14	91.51	68.94	9.86	247.57	50.11	2.59	4.68	57.39	40.66	42.83	33.85	35.74	2.18	155.27	12.86	8.29	4.64	0.39	26.19	20.05	0.57	20.61	0.76	0.35	2.57	0.09	3.77
ML1-bottle3	30	6.7	42.48	28.18	92.26	67.97	9.88	247.45	50.17	2.59	4.69	57.45	40.71	42.88	33.89	35.78	2.19	155.45	12.87	8.3	4.65	0.39	26.22	20.07	0.57	20.64	0.76	0.35	2.57	0.09	3.77
ML1-bottle1	75	6.99	46.28	28.51	90.88	59.56	10.31	242.32	47.94	2.52	4.54	54.99	35.28	44.15	33.82	37.84	1.95	152.11	13.82	8.34	3.88	0.41	26.24	19.39	0.6	19.99	0.66	0.37	2.71	0.09	3.81
ML1-bottle2	75	6.45	40.92	27.14	90.74	67.45	9.51	242.1	42.03	2.6	4.11	48.66	32.56	41.61	31.21	35.73	1.8	141.82	12.72	7.7	3.58	0.38	24.18	17.89	0.55	18.43	0.6	0.37	2.41	0.08	3.44
ML1-bottle3	75	6.36	40.38	25.96	90.19	67.16	9.39	239.18	42.9	2.49	4.14	49.48	32.13	41.07	30.8	34.4	1.77	139.24	12.57	7.6	3.53	0.38	23.88	17.66	0.55	18.2	0.67	0.36	2.38	0.09	3.49
ML2-bottle1	30	4.01	21.84	8.13	64	45.51	6.85	150.34	55.18	1.96	5.29	62.44	43.18	43.95	33.58	35.98	2.18	158.89	11.49	8.55	5.36	0.29	25.7	22.67	0.55	23.23	0.79	0.37	2.69	0.1	3.94
ML2-bottle2	30	3.86	21.07	7.84	61.6	43.8	6.6	144.78	53.22	1.89	5.1	60.21	41.65	42.39	32.39	34.7	2.11	153.23	11.08	8.25	5.17	0.28	24.78	21.87	0.53	22.4	0.76	0.36	2.59	0.1	3.81
ML2-bottle3	30	3.98	21.69	8.07	59.7	42.45	6.8	142.7	54.8	1.95	5.25	62	42.88	43.65	33.35	35.73	2.17	157.77	11.41	8.49	5.33	0.29	25.52	22.51	0.55	23.06	0.78	0.37	2.67	0.1	3.92
ML2-bottle1	75	3.89	15.89	5.62	48.09	38.57	6.52	118.58	53.7	2.14	5.36	61.2	41.35	41.4	32.9	35.2	2.23	153.08	11.95	8.35	5.45	0.36	26.1	22.15	0.6	22.75	0.83	0.37	2.67	0.1	3.96
ML2-bottle2	75	3.64	14.9	5.27	45.11	36.18	6.12	111.23	50.37	2.01	5.03	57.41	38.79	38.83	30.86	33.02	2.09	143.58	11.21	7.83	5.11	0.34	24.48	20.78	0.56	21.34	0.78	0.34	2.5	0.09	3.72
ML2-bottle3	75	3.61	14.76	5.22	44.68	35.84	6.06	110.17	49.89	1.99	4.98	56.86	38.42	38.46	30.57	32.7	2.07	142.22	11.1	7.75	5.06	0.33	24.25	20.58	0.55	21.13	0.77	0.34	2.48	0.09	3.68
ML3-bottle1	30	2.9	20.63	5.96	29.06	22.96	3.93	85.43	55.78	1.97	5.48	63.23	42.08	41.25	30.29	33.03	1.99	148.63	8.97	8.07	5.67	0.15	22.87	23.51	0.49	24	0.75	0.36	2.58	0.1	3.78
ML3-bottle2	30	2.81	19.99	5.77	28.17	22.25	3.81	82.8	54.06	1.91	5.31	61.28	40.78	39.98	29.36	32.01	1.93	144.06	8.69	7.82	5.5	0.15	22.16	22.79	0.47	23.26	0.72	0.35	2.5	0.1	3.67
ML3-bottle3	30	2.87	20.44	5.9	28.8	22.75	3.89	84.66	55.28	1.95	5.43	62.66	41.7	40.88	30.02	32.73	1.97	147.3	8.89	8	5.62	0.15	22.66	23.3	0.48	23.78	0.74	0.36	2.55	0.1	3.75
ML3-bottle1	75	2.44	15.96	4.98	21.28	18.48	3.46	66.6	54.4	2.39	5.47	62.26	39.7	37.5	30	32.6	2.14	141.94	10.3	7.92	5.99	0.3	24.51	23.1	0.59	23.69	0.86	0.37	2.61	0.1	3.93
ML3-bottle2	75	2.39	15.69	4.9	20.92	18.17	3.4	65.47	53.48	2.34	5.47	61.29	39.03	36.86	29.49	32.05	2.1	139.53	10.12	7.79	5.89	0.3	24.09	22.71	0.58	23.29	0.85	0.36	2.57	0.09	3.86
ML3-bottle3	75	2.41	15.8	4.93	21.07	18.3	3.42	65.93	53.86	2.36	5.41	61.63	39.3	37.13	29.7	32.27	2.12	140.52	10.2	7.84	5.93	0.3	24.27	22.87	0.59	23.45	0.85	0.36	2.58	0.09	3.89
ML4-bottle1	30	2.31	11.95	3.33	13.63	11.69	2.59	45.49	52.23	2.56	5.56	60.34	27.24	27	21.05	20.28	1.48	97.06	9.29	7.12	5.38	0.22	22.02	21.91	0.54	22.45	0.85	0.28	2.21	0.1	3.44
ML4-bottle2	30	2.29	11.82	3.29	13.48	11.57	2.56	45.01	51.68	2.53	5.5	59.7	26.96	26.72	20.83	20.07	1.47	96.03	9.19	7.05	5.32	0.22	21.78	21.68	0.53	22.21	0.84	0.28	2.19	0.09	3.4

ML4-bottle3	30	2.26	11.66	3.25	13.3	11.41	2.53	44.4	50.97	2.69	5.43	59.09	26.59	26.35	20.54	19.79	1.45	94.73	9.07	6.95	5.25	0.22	21.49	21.38	0.53	21.91	0.83	0.27	2.16	0.09	3.35
ML4-bottle1	75	1.72	9.87	3.52	9.73	8.96	2.12	35.92	51.9	2.61	5.48	59.99	24	21.5	17.2	18.7	1.22	82.62	8.26	6.29	4.86	0.15	19.56	22.5	0.55	23.05	0.84	0.28	2.24	0.09	3.45
ML4-bottle2	75	1.57	9.05	3.23	8.92	8.21	1.94	32.92	47.57	2.48	5.02	55.08	22	19.71	15.77	17.14	1.12	75.73	7.57	5.77	4.45	0.13	17.93	20.62	0.5	21.13	0.77	0.26	2.05	0.08	3.17
ML4-bottle3	75	1.7	9.78	3.49	9.64	8.88	2.1	35.59	51.43	2.69	5.43	59.55	23.78	21.31	17.05	18.53	1.21	81.88	8.19	6.23	4.82	0.15	19.38	22.3	0.54	22.84	0.83	0.28	2.22	0.09	3.42
ML5-bottle1	30	1.19	7.2	2.44	5.95	4.66	1.31	22.75	57.79	2.26	5.55	65.6	12.41	13.67	11.13	19.15	0.64	57	3.58	6.93	3.66	0.04	14.21	22.72	0.47	23.19	0.76	0.18	1.78	0.05	2.77
ML5-bottle2	30	1.17	7.08	2.4	5.85	4.58	1.29	22.36	56.81	2.23	5.45	64.49	12.2	13.44	10.94	18.83	0.63	56.03	3.51	6.82	3.6	0.04	13.97	22.33	0.47	22.8	0.74	0.18	1.75	0.05	2.72
ML5-bottle3	30	1.14	6.86	2.32	5.66	4.43	1.25	21.66	55.02	2.16	5.28	62.45	11.82	13.01	10.59	18.23	0.61	54.27	3.4	6.6	3.48	0.04	13.53	21.63	0.45	22.08	0.72	0.17	1.7	0.05	2.64
ML5-bottle1	75	0.88	6.33	2.32	4.68	3.76	1.04	19.01	56.3	2.7	5.43	64.43	11.1	11.2	8.77	13.1	0.55	44.72	3.31	5.21	3.54	0.06	12.12	21.8	0.51	22.31	0.85	0.2	2.02	0.07	3.14
ML5-bottle2	75	0.85	6.18	2.27	4.58	3.67	1.02	18.57	55.01	2.63	5.31	62.94	10.84	10.94	8.57	12.8	0.54	43.69	3.23	5.09	3.46	0.06	11.84	21.3	0.5	21.79	0.83	0.2	1.97	0.06	3.06
ML5-bottle3	75	0.81	5.89	2.16	4.36	3.5	0.97	17.7	52.42	2.51	5.06	59.98	10.33	10.43	8.16	12.2	0.51	41.63	3.08	4.85	3.3	0.06	11.28	20.3	0.47	20.77	0.79	0.19	1.88	0.06	2.92
ML6-bottle1	30	0.95	5.04	1.54	5.99	6.79	1.23	21.53	59.8	2.56	5.61	67.97	6.27	7.92	6.3	4.35	0.37	25.21	1.93	4.75	3.27	0.03	9.98	22.72	0.62	23.34	0.99	0.19	1.75	0.06	2.98
ML6-bottle2	30	0.93	4.94	1.51	5.87	6.65	1.2	21.1	58.61	2.51	5.5	66.61	6.15	7.76	6.17	4.26	0.37	24.71	1.89	4.65	3.21	0.03	9.78	22.26	0.61	22.87	0.97	0.18	1.72	0.06	2.93
ML6-bottle3	30	0.86	4.59	1.4	5.46	6.19	1.12	19.62	54.48	2.33	5.11	61.92	5.71	7.21	5.74	3.96	0.34	22.97	1.75	4.33	2.98	0.03	9.09	20.69	0.57	21.26	0.9	0.17	1.6	0.05	2.72
ML6-bottle1	75	0.7	4.48	1.55	2.88	2.43	0.74	12.78	58	2.48	5.56	66.04	5.29	6.2	5.58	5.16	0.25	22.48	1.41	4.82	2.79	0.03	9.05	23	0.56	23.56	0.89	0.17	1.58	0.05	2.69
ML6-bottle2	75	0.61	3.94	1.37	2.53	2.14	0.65	11.25	56.84	2.43	5.45	64.71	4.66	5.46	4.91	4.54	0.22	19.79	1.24	4.24	2.46	0.03	7.96	20.24	0.49	20.73	0.78	0.15	1.39	0.04	2.36
ML6-bottle3	75	0.69	4.44	1.54	2.85	2.4	0.73	12.65	57.42	2.45	5.5	65.37	5.24	6.14	5.52	5.11	0.25	22.26	1.4	4.77	2.76	0.03	8.96	22.77	0.55	23.32	0.88	0.17	1.56	0.05	2.66

Abbreviations: MI1, MI2 and MI3, three bottles of initial wine without exogenous acetaldehyde at 0 days; MiE1, MiE2 and MiE3 (i=1, 2, 3,4 5, 6), three bottles of wine with different initial acetaldehyde (1-6) at 30 days of aging; MiH1, MiH2 and MiH3 (i=1, 2, 3,4 5, 6), three bottles of wine with different initial acetaldehyde (1-6) at 75 days of aging; M1, wines containing 7.86±0.10 mg/L acetaldehyde; M2, wines containing 41.72±1.97 mg/L acetaldehyde; M3, wines containing 61.19±3.93 mg/L acetaldehyde; M4, wines containing 128.10±3.08 mg/L acetaldehyde; M5, wines containing 203.02±6.00 mg/L acetaldehyde; M6, wines containing 259.02±4.95 mg/L acetaldehyde. CND, cyanidin-3-glucoside; DPD, delphinidin-3-glucoside; DPDR, delphindin 3-O rutinoside; MVD, malvidin-3-glucoside; MVDAC, malvidin-3-glucoside acetate; PND, peonidin-3-glucoside; GA, gallic acid; VA, vanillic acid; TPCA, trans-p-coumaric acid; TBA, total benzoic acid; PDB1, procyanidin b1; CCN, catechin; ECCA, epicatechin; PDB2, procyanidin b2; EGCN, epigallocatechin; MCN, myricetin; QCN, quercetin; KPL, kaempferol; CFA, caffeic acid; THMCA, trans-ferulic acid; TCA, total hydroxycinnamic acid; PDN, polydatin; CRVTL, cis-resveratrol; TRVTL, trans-resveratrol; VFN, viniferin; TS, total stilbenes.
